# Decreased Heart Rate Variability in COVID-19

**DOI:** 10.1007/s44231-022-00024-1

**Published:** 2022-12-01

**Authors:** Chengfen Yin, Jianguo Li, Zhiyong Wang, Yongle Zhi, Lei Xu

**Affiliations:** 1 Department of Critical Care Medicine, Tianjin Third Central Hospital, Tianjin Key Laboratory of Extracorporeal Life Support for Critical Diseases, Artificial Cell Engineering Technology Research Center, Tianjin Institute of Hepatobiliary Disease, Tianjin, China; 2 Department of Respiratory and Critical Medicine, Tianjin Haihe Hospital, Tianjin, China

**Keywords:** Angiotensin converting enzyme 2, Coronavirus disease 2019, Prognosis, Tachycardia, Temperature, Myocardium, Heart rate variability

## Abstract

**Purpose:**

Coronavirus disease 2019 (COVID-19) is caused by severe acute respiratory syndrome coronavirus 2 (SARS-CoV-2), which primarily infects the lower airways and binds to angiotensin-converting enzyme 2 (ACE2) on alveolar epithelial cells. ACE2 is widely expressed not only in the lungs but also in the cardiovascular system. Therefore, SARS-CoV-2 can also damage the myocardium. This report aimed to highlight decreased heart rate variability (HRV) and cardiac injury caused by SARS-CoV-2.

**Materials and Methods:**

We evaluated three COVID-19 patients who died. Patients’ data were collected from electronic medical records. We collected patient’s information, including baseline information, lab results, body temperature, heart rate (HR), clinical outcome and other related data. We calculated the HRV and the difference between the expected and actual heart rate changes as the body temperature increased.

**Results:**

As of March 14, 2020, 3 (2.2%) of 136 patients with COVID-19 in Tianjin died in the early stage of the COVID-19 epidemic. The immediate cause of death for Case 1, Case 2, and Case 3 was cardiogenic shock, cardiac arrest and cardiac arrest, respectively. The HRV were substantially decreased in the whole course of all three cases. The actual increases in heart rate were 5 beats/min, 13 beats/min, and 4 beats/min, respectively, less than expected as their temperature increased. Troponin I and Creatine Kinase MB isoenzyme (CK-MB) were substantially increased only in Case 3, for whom the diagnosis of virus-related cardiac injury could not be made until day 7. In all three cases, decreased in HRV and HR changes occurred earlier than increases in cardiac biomarkers (e.g., troponin I and CK-MB).

**Conclusions:**

In conclusion, COVID-19 could affect HRV and counteract tachycardia in response to increases in body temperature. The decreases of HRV and HR changes happened earlier than the increases of myocardial markers (troponin I and CK-MB). It suggested the decreases of HRV and HR changes might help predict cardiac injury earlier than myocardial markers in COVID-19, thus its early identification might help improve patient prognosis.

**Supplementary Information:**

The online version contains supplementary material available at 10.1007/s44231-022-00024-1.

## Introduction

In December 2019, an outbreak of a novel coronavirus, now formally named severe acute respiratory syndrome coronavirus 2 (SARS-CoV-2), emerged in Wuhan, Hubei Province, Central China. SARS-CoV-2 causes coronavirus disease 2019 (COVID-19) that has rapidly spread worldwide through close human interactions or the spilled respirational material (cough, sneeze) of the infected people. Accordingly, the World Health Organization officially announced the COVID-19 outbreak as a pandemic on March 12th, 2020 [1].

Information about the new coronavirus and its health impact is constantly being updated. SARS-Cov-2 predominantly infects the lower airways and binds to angiotensin-converting enzyme 2 (ACE2) on alveolar epithelial cells. ACE2 is widely expressed in the human body not only in the lungs, but also in the cardiovascular system, thus SARS-CoV-2 can also infect human cardiomyocytes and exert cytotoxic effects [2]. However, the pathophysiological mechanisms underlying myocardial injury caused by COVID-19 are not well known so far. The possible mechanisms could be direct systemic inflammation, exaggerated cytokine response, hypoxia, disseminated intravascular coagulation, myocardial fibrosis, and direct damage to the cardiomyocytes [2, 3]. Patients with COVID-19 with acute myocardial injury have higher probability of death [4]. Therefore, early detection of cardiovascular damage caused by COVID-19 is important [5]. Although the cardiomyocyte structural protein, troponin I, level in plasma is a known indicator of cardiac injury. Heart rate variability (HRV) has been widely used for decades to quantify risk in a wide variety of both cardiac and noncardiac disorders [6].

The report aimed to highlight autonomic nervous system dysfunction and cardiac injury caused by SARS-CoV-2. We present three COVID-19 infections that resulted in death. We found that COVID-19 could affect coupling between the autonomic nervous system (ANS) and the sinus node, thus affecting HRV and counteract tachycardia in response to increases in body temperature.

## Case Presentation

This study was approved by the National Health Commission of China and Ethics Commission of Tianjin Third Central Hospital (2020-03-14). The need for written informed consent was waived by the Ethics Commission of the hospital because of the COVID-19 pandemic.

As of March 14, 2020, a total of 136 patients have been diagnosed with COVID-19 in Tianjin, China. Of them, 3 (2.2%) (2 female and 1 male) died in the early stage of the COVID-19 epidemic, and they were aged 64, 65 and 74 years, respectively. All three cases were diagnosed and treated according to the New Coronavirus Pneumonia Prevention and Control Program (5th edition) [7]. The demographic and clinical characteristics and laboratory test results of these cases on admission are shown in Supplemental Digital Content-Table 1. According to the New Coronavirus Pneumonia Prevention and Control Program (5th edition) [7], COVID-19 is classified into four types, namely mild type, moderate type, severe type, and critical type. Mild type represents mild clinical symptoms without abnormal chest imaging findings. Moderate type is defined as having both clinical symptoms and abnormal chest imaging findings. Patients are diagnosed as severe type when the disease progresses to meet any of the following criteria: (a) respiratory distress with respiration rate ≥ 30 breaths/min; (b) oxygen saturation ≤ 93% in resting state; or (c) PaO_2_/FiO_2_ (partial pressure of oxygen/fraction of inspired oxygen) ≤ 300 mmHg (1 mmHg = 0.133 kPa). Critical type occurs when the disease progresses rapidly with any of the following conditions: (a) respiratory failure requiring mechanical ventilation; (b) shock; or (c) other organ failure requiring intensive care unit admission for monitoring and treatment. On admission, the severity of COVID-19 was categorized as moderate type in Case 1 and Case 3 and as severe type in Case 2. All three cases had at least one comorbidity (e.g., hypertension, diabetes mellitus, and cardiovascular disease), varying degrees of cough and fever on admission, and bilateral lung ground-glass opacity on computed tomography imaging. The immediate cause of death for Case 1, Case 2, and Case 3 was cardiogenic shock (hospital stay, day 2), cardiac arrest (hospital stay, day 8), and cardiac arrest (hospital stay, day 9), respectively (Supplemental Digital Content-Table 1).

**Table 1:** Cardiac markers

Case	Cardiac markers	Day 1	Day 2	Day 3	Day 4	Day 5	Day 6	Day 7	Day 8	Day 9	Normal ranges
Case 1	Myoglobin (ug/l)	69.3	78.5								0–61.5
	Troponin I (ug/ml)	0.045	0.061								0–0.12
	CK-MB (ug/l)	0.35	0.52								0–5.31
	NTpro-BNP (pg/ml)	1190	NA								0–900
	SDNN (ms)	31.73	NA								60–276
Case 2	Myoglobin (ug/l)	79.2	50.1	39.3	NA	40.2	32.6	28.6	NA		0–61.5
	Troponin I (ug/ml)	0.012	0.012	0.012	NA	0.012	0.012	0.012	NA		0–0.12
	CK-MB (ug/l)	1.04	1.07	0.99	NA	0.77	0.39	0.22	NA		0–5.31
	NTpro-BNP (pg/ml)	106	265	600	NA	62	139	215	113		0–900
	SDNN (ms)	29.00		66.933	41.952		44.572	26.443	21.908		60–276
Case 3	Myoglobin (ug/l)	50.3	NA	NA	NA	97.1	402.7	2000	51.5	1598	0–61.5
	Troponin I (ug/ml)	0.012	NA	NA	NA	0.016	0.015	0.039	0.012	1.16	0–0.12
	CK-MB (ug/l)	0.27	NA	NA	NA	0.83	1.37	8.02	0.33	0.86	0–5.31
	NTpro-BNP (pg/ml)	NA	NA	NA	NA	853	831	NA	83	NA	0–900
	SDNN (ms)	45.607			37.238		35.989	32.660		28.284	60–276

*NA* not available

**Fig. 1:**
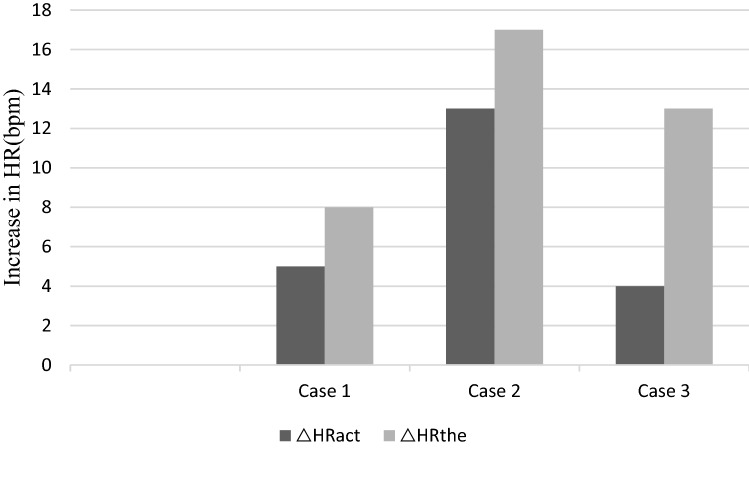
The actual increase and theoretical increase in heart rate as body temperature increases. *△HR*_*act*_ The actual increase in heart rate as the body temperature increases, *△HR*_*the*_ Theoretical increase in heart rate as body temperature increases

The most common HRV measures are the standard deviation of all NN intervals (SDNN) and the squares of differences between adjacent NN intervals (rMSSD) [8]. Several studies have specifically investigated the validity and reproducibility of ultra-short HRV measurements [9, 10]. HRV measures in this study were SDNN from 10 s’ ECG. Before HRV analysis the ECG data were visually inspected to exclude non-sinus rhythm, ectopic beats, and artifacts, such as premature ventricular beats, electrical ‘noise’, or aberrant beats. The SDNNs were substantially decreased, indicated a reduction in HRV, in the whole course of all three cases. In all three cases, decreases in HRV occurred earlier than increases in cardiac biomarkers as listed in Table 1. The changes in heart rate and body temperature are presented in Table 2, Fig. 1. Heart rates at the maximum body temperature and at minimum body temperature within one day were selected based on the records in temperature sheet. The heart rates of Case 1, Case 2, and Case 3 should increase by 8 beats/min, 17 beats/min, and 13 beats/min, respectively, as their temperature increased. However, the actual increases in heart rate were only 5 beats/min, 13 beats/min, and 4 beats/min, respectively. In all three cases, decreases in HR changes occurred earlier than increases in cardiac biomarkers (e.g., troponin I and CK-MB), same as HRV.

**Table 2:** Changes in heart rate and body temperature

Case	*T*_min_ day 1 (℃)	HR_Tmin_ (bpm)	*T*_max_ day 1 (℃)	HR_Tmax_ (bpm)	△HR_act_ (bpm)	△HR_the_ (bpm)	△HR_the-act_ (bpm)
Case 1	37.3	86	38.5	91	5	8	3
Case 2	36	79	38.7	92	13	17	4
Case 3	36.4	78	38.5	82	4	13	9

*T*_*min*_ Minimum body temperature, *HR*_*Tmin*_ Heart rate at the minimum body temperature, *T*_*max*_ Maximum body temperature, *HR*_*Tmax*_ Heart rate at the maximum body temperature, *△HR*_*act*_ The actual increase in heart rate as the body temperature increases, *△HR*_*the*_ Theoretical increase in heart rate as body temperature increases, *△HR*_*the-act*_ The difference between the theoretical and actual increase of heart rate

The cardiac biomarkers are shown in Table 1. COVID-19-related cardiac injury can be diagnosed if serum levels of cardiac biomarkers (e.g., troponin I) are above the 99th percentile upper reference limit [11]. Troponin I and CK-MB were substantially increased only in Case 3, for whom the diagnosis of virus-related cardiac injury could not be made until day 7. Myoglobin levels were above the normal limit on admission in Case 1 and Case 2. Myoglobin levels were above the normal limit from day 5 to day 9 in Case 3. NT-proBNP levels were above the normal limit in Case 1, but within normal in Case 2 and Case 3 for the duration of the hospital stay.

## Discussion

Although the clinical manifestations of COVID-19 are predominantly respiratory symptoms, some patients have severe cardiovascular damage [11], and some patients with underlying cardiovascular diseases might have an increased mortality risk [11]. Therefore, understanding the effect of SARS-CoV-2 to the cardiovascular system and the underlying mechanisms is of great importance as it will allow for timely and effective treatment.

The sinus node is the natural pacemaker of the heart and possesses its own intrinsic activity; nevertheless, various external and internal stimuli that change the balance between vagal and sympathetic tone influence the final basic heart rate. HRV is considered an indirect measure of autonomic regulation of cardiac activity. It can also reflect the coupling between the ANS and the sinoatrial node [12]. Studying the physiological indicators (e.g., HR) in critically ill patients help to identify underlying conditions related to inherent dynamics and overall variability within a time series [13]. Therefore, HRV has become an important and well-recognized tool in identifying patients at risk of cardiogenic death [14]. Significant autonomic dysregulation can also be seen in patients with COVID-19, manifested by decreased activity of the sympathetic nervous system, along with the increase in the parasympathetic component [15]. Thus, the ANS is responsible for the regulation of this inflammatory reflex, and its balance is essential to the maintenance of the body’s homeostasis [16, 17]. In the three present cases, decreases in HRV occurred earlier than changes in cardiac markers. In future similar cases, staying alert to early decrease in HRV may help predict the occurrence of cardiac events in the early stage.

The cause of reduced HRV in infectious disease remains unclear, but two theories have been proposed. The first theory focuses on reduction of vagal tone, while the second theory stipulates that normal physiology has fractal-like properties with high levels of complexity, explaining reduced HRV [12]. Severe disease reflects a “decomplexification” that can mainly be attributed to uncoupling between different restorative mechanisms [12]. Accumulating evidence supports a potential third mechanism associated with an intracardiac origin of HRV, which was first proposed by Griffin et al. [12]. According to this hypothesis, sinus node cells with an extreme heterogeneity in electrophysiological properties and intercellular connections of sinus node cells can be viewed as an amplifier of various input signals. In infectious or cardiovascular diseases, an unfavorable metabolic milieu could affect ion channel gating properties or membrane receptor densities, with significant impact on the level and variability of pacemaker activity. In addition, a possible reduced responsiveness of sinus node cells to external stimuli could also negatively affect HRV [12].

In general, heart rate has a specific relationship with body temperature. As body temperature increases, heart rate will also increase [18]. Recent study has shown that in acutely admitted patients, the heart rate will increase by 6.4 beats/min when body temperature increases 1 °C than the normal limit after adjusting for age, oxygen saturation, and mean blood pressure [18]. As such, the heart rates of Case 1, Case 2, and Case 3 should have increased by 8 beats/min, 17 beats/min, and 13 beats/min, respectively, as their temperature increased. However, the actual increases in heart rate were only 5 beats/min, 13 beats/min, and 4 beats/min, respectively. Increases in the body temperature of the three present cases did not correspond with appropriate increases in heart rate. The difference between the expected and actual heart rate indicates a decrease in the patients’ heart rate changes, which could be due to a problem in coupling between the ANS and the sinus node. ANS imbalance, with a shift toward decreased vagal and increased sympathetic tone, has been proven to be associated with higher risk of cardiac mortality. The presence of autonomic dysfunction should alert clinicians to distinguish the possibility of coexisting cardiovascular risk factors. Early detection of the preclinical phase of cardiac autonomic dysfunction may enable more aggressive treatment and control of cardiovascular risk factors. Some data indicate that autonomic imbalance can be related to an increased risk of arrhythmias, and even sudden future death [14].

## Conclusions

In conclusion, COVID-19 could affect HRV and counteract tachycardia in response to increases in body temperature. The decreases of HRV and HR changes happened earlier than myocardial markers (troponin I and CK-MB). It suggested the decreases of HRV and HR changes might help predict cardiac injury earlier than myocardial markers in COVID-19, thus its early identification might help improve patient prognosis.

## Supplementary Information

Below is the link to the electronic supplementary material.

Supplementary file1 (DOCX 18 KB)

## Data Availability

All data generated or used during the study appear in the submitted article.

## Abbreviations

ACE2: Angiotensin-converting enzyme 2
ANS: Autonomic nervous system
COVID-19: Coronavirus disease 2019
HRV: Heart rate variability
SARS-CoV-2: Severe acute respiratory syndrome coronavirus 2
HR: Heart rate
CK-MB: Creatine Kinase MB isoenzyme
