# Supplementary material for: Decreased Heart Rate Variability in COVID-19
Source: Intensive Care Res. 2022 Dec 1;3(1):87–91. doi: 10.1007/s44231-022-00024-1 (PMC9713139; doi:10.1007/s44231-022-00024-1)
Supplement: Supplementary file 1 — Supplementary file1 (DOCX 18 KB) [file 44231_2022_24_MOESM1_ESM.docx]

**Supplemental Digital Content-Table 1. Clinical demographic characteristics and laboratory results on admission**

| Characteristics | Case 1 | Case 2 | Case 3 | Normal ranges |
| --- | --- | --- | --- | --- |
| Age (years) | 65 | 64 | 74 |  |
| Sex | Female | Female | Male |  |
| Height (cm) | 150 | 165 | 178 |  |
| Weight (kg) | 66 | 75 | 86 |  |
| BMI (kg/m^2^) | 29.33 | 27.55 | 27.14 |  |
| Medical history | Hypertension, DM, CAD, CABG | CAD | Hypertension, DM, cerebral infarction, right renal cell carcinoma, right nephrectomy |  |
| Smoking | No | No | No |  |
| Alcohol | No | No | No |  |
| Vital signs (on admission) | | | |  |
| HR (bpm) | 88 | 94 | 76 |  |
| RR (breath/min) | 23 | 36 | 21 |  |
| Body temperature (℃) | 37.4 | 38.3 | 38.4 |  |
| SBP (mmHg) | 141 | 114 | 159 |  |
| DBP (mmHg) | 80 | 84 | 86 |  |
| MAP (mmHg) | 100 | 94 | 110 |  |
| SPO_2_ (%) | 100 | 98 | 100 |  |
| Others | | | |  |
| Q-SOFA score | 0 | 1 | 1 |  |
| CURB-65 score | 0 | 1 | 0 |  |
| APACHE II | 5 | 15 | 11 |  |
| Hospital stay (day) | 2 | 8 | 9 |  |
| Cause of death | Cardiogenic shock | Cardiac arrest | Cardiac arrest |  |
| Blood gas | | | | |
| PH | 7.44 | 7.45 | 7.4 | 7.35-7.45 |
| PaO_2_ (mmHg) | 113 | 108 | 124 | 80-100 |
| PaCO_2_ (mmHg) | 24.4 | 31.1 | 39.1 | 35-45 |
| FiO_2_ (%) | 29 | 29 | 29 | 21 |
| Complete blood count | | | | |
| White blood cell (*10^9^/l) | 4.59 | 3.84 | 10.98 | 4-10 |
| Hemoglobin (g/l) | 111 | 123 | 129 | 120-160 |
| Platelet (*10^9^/l) | 190 | 90 | 185 | 100-400 |
| Lymphocyte (*10^9^/l) | 1.7 | 1.0 | 1.2 | 0.8-4 |
| Electrolyte | | | |  |
| Na^+^ (mmol/l) | 135 | 133 | 137 | 135-145 |
| K^+^ (mmol/l) | 4.6 | 3.5 | 4.3 | 3.5-5.5 |
| Liver function | | | | |
| AST (U/L) | 26 | 64 | 54 | 17-59 |
| ALT (U/L) | 21 | 22 | 22 | 21-72 |
| Renal function (on admission) | | | | |
| Serum creatinine (µmol/l) | 84 | 45 | 87 | 58-110 |
| BUN (mmol/l) | 12 | 4 | 6 | 3.2-7.1 |
| Other lab results | | | | |
| CRP (mg/l) | 85.3 | 2.94 | 54.2 | 0-10 |
| PCT (ng/ml) | 0.058 | 0.04 | 0.047 | 0-0.5 |

Note: BMI=body mass index, DM=diabetes mellitus, CAD=Cardiovascular disease, CABG=Coronary artery bypass surgery, HR=Heart rate, RR=Respiratory frequency, SBP=Systolic blood pressure, DBP=Diastolic blood pressure, Q-SOFA score=Quick-sequential organ failure assessment, APACHE II=Acute physiological and chronic health status evaluation II, PaO_2_=Arterial oxygen partial pressure, PaCO_2_=Arterial partial pressure of carbon dioxide, FiO_2_=fraction of inspired oxygen, Na^+^=Sodium ion, K^+^=Potassium ion, AST=Aspartate aminotransferase, ALT=Alanine aminotransferase, BUN=Blood urea nitrogen, CRP=C-reactive protein, PCT=Procalcitonin.
